# Correlates of telomere length shortening in peripheral leukocytes of HIV-infected individuals and association with leukoaraiosis

**DOI:** 10.1371/journal.pone.0218996

**Published:** 2019-06-27

**Authors:** Rumi Minami, Soichiro Takahama, Masahiro Yamamoto

**Affiliations:** Internal Medicine, Clinical Research Institute, National Hospital Organization, Kyushu Medical Center, Fukuoka, Japan; Rush University, UNITED STATES

## Abstract

Telomere length (TL) is a marker of cellular and biological aging. Human immunodeficiency virus (HIV) infection has been reported to be associated with short TLs, which suggests that accelerated biological aging occurs in some cellular compartments of HIV^+^ individuals. In this study, we measured the TLs of peripheral leukocytes of HIV^+^ and healthy individuals and examined the biological and environmental correlates of TL. We also investigated the influence of TL on leukoaraiosis, an indicator of cerebral small vessel disease, in HIV^+^ individuals. Three hundred and twenty-five HIV^+^ individuals who received stable combination antiretroviral therapy (cART) for >1 year and achieved viral loads of <40 RNA copies/mL were enrolled along with 147 healthy individuals. Relative TLs of leukocytes were estimated by quantitative real-time polymerase chain reaction. Leukoaraiosis was assessed in 184 HIV^+^ individuals by fluid-attenuated inversion recovery magnetic resonance imaging. We analyzed several covariates, including markers of HIV infection, cART, and social/environmental factors; variables associated with TL length in univariate analyses were incorporated into multivariate models. The TLs of peripheral leukocytes of HIV^+^ individuals were significantly shorter than those of healthy individuals, and the rate of LT length decline with increasing age was greater. Linear regression analysis showed that in HIV^+^ individuals, increasing age, cART without integrase-stand transfer inhibitors (INSTI), failure to achieve viral loads of <40 copies/mL within 1 year of initiating cART, and substance use were significantly associated with shorter TLs, even after adjustment for the effects of age. Logistic regression analysis indicated an increasing risk of leukoaraiosis was associated with older age, shorter TLs, hypertension, and carotid artery plaque. Multivariate regression analysis indicated that older age and shorter TLs were significant risk factors for leukoaraiosis. In summary, our data showed that TL shortening in HIV^+^ individuals was independently associated with leukoaraiosis, and was associated with age, control of viral loads, use of INSTI, and substance use. Our results suggest that effective viral control and less toxic cART can help reduce TL shortening and improve outcomes among HIV^+^ individuals.

## Introduction

HIV^+^ individuals are at increased risk of age-related non- acquired immunodeficiency syndrome (AIDS) morbidity and mortality compared with healthy individuals. In recent years, premature aging (i.e., non-chronological biological aging mediated by accelerated cellular senescence) has become an important issue in HIV infection [1]. To bridge the gap between chronological and biological age, effective and validated biomarkers of aging are needed to predict the health outcomes of individuals with HIV, especially in the elderly.

Telomeres are specialized structures at the ends of chromosomes that undergo shortening with each cell division, subsequently leading to telomere loss and the end replication problem in actively-dividing tissues [2]. In most inactive tissues, cellular environmental factors such as inflammation and oxidative stress are important factors contributing to telomere loss [3]. Measures of telomere length (TL) have shown synchronicity across tissues in the same individual [4,5]. For this reason, TL is an indicator of cellular senescence [6] and is considered an effective and validated biomarker of aging. TL shortening is associated with a wide range of conditions including cardiovascular disease [7], renal dysfunction [8], osteoporosis [9], cancer [10], dementia [11] and some other age-related diseases [12,13]. HIV infection has been reported to be associated with short TLs. During HIV infection several factors, such as chronic immune activation, inflammation, oxidative stress, HIV proteins, and cART, may contribute to premature TL shortening [10,14,15]. However, previous studies have reported conflicting findings as to which factors are essential for TL shortening, potentially due to differences among study subjects. Thus, in this study we sought to investigate leukocyte TL (LTL) in cART-treated HIV^+^ individuals who achieved viral control and to identify the factors associated with shorter LTLs in these individuals.

Leukoaraiosis is a typical marker of cerebral small vessel disease, which is one of the most common degenerative vessel disorders of the aging brain. Leukoaraiosis has been reported to be associated with both HIV infection [16] and advancing age [17], and represents several different changes in white matter such as partial loss of myelin, oligodendroglia and axons, fibrohyalinosis, activation of macrophages, and dilated perivascular spaces [18,19]. Leukoaraiosis can be measured by examining white matter hyperintensity (WMH) on fluid attenuated inversion recovery (FLAIR) images obtained from magnetic resonance imaging (MRI). Leukoaraiosis is associated with increased risk of stroke, cognitive and functional impairment, dementia, and death [20], all of which are involved in age-related vascular risk factors. Therefore, leukoaraiosis is an indicator of biological aging of the brain [21]. Recently, as more people are living longer with HIV, HIV-Associated Neurocognitive Disorder (HAND) has become an important issue. Inflammation caused by HIV infection contributes to HAND pathogenesis and is associated with WMH and brain atrophy. Several vascular risk factors have been studied as potential predictors of HIV associated neurocognitive complications [22]. In this study, we also investigated the relationship between leukoaraiosis and LTL and examined the usefulness of LTL as a biomarker of brain aging.

## Materials and methods

### Ethics statement

This study was approved by the Ethics Committee of Kyushu Medical Center (ethics reference number: 11–95) and conformed to the principles laid out in the Declaration of Helsinki. All study participants provided written informed consent.

### Study participants

Three hundred and twenty-five chronically HIV^+^ individuals on stable cART for >1 year who achieved viral loads <40 RNA copies/mL were enrolled, and 147 age/sex frequency matched HIV-seronegative healthy volunteers were recruited among the employees of Kyushu Medical Center. Sociodemographic information of participants is shown in Table 1. The following criteria were evaluated as covariates in the analyses: hypertension as defined by prior physician’s diagnosis; systolic blood pressure >140 mmHg, diastolic blood pressure >90 mmHg, or the use of antihypertensive drugs; and substance abuse, defined by both past history and current use of any illicit/controlled substance.

**Table 1:** Baseline characteristics of study participants.

	HIV-1 positives	Controls	p value
Number of subjects	325	147	
Sex (Male/Female)	314/11	138/9	0.1845^b^
Age (range)	39.0 (18–69)	39.5 (22–63)	0.8281^a^
BMI (range) (kg/m^2^)	22.7 (12.8–36.7)	23.0 (17.2–31.3)	0.3211^a^
Smokers (yes/no) (past history) (%)	178/147 (54.7%)	68/79 (46.3%)	0.0865^b^
Smokers (yes/no) (current) (%)	121/204(37.2%)	42/105 (28.6%)	0.0645^b^
Hypertension n (%)	55 (16.9%)	29 (19.7%)	0.4639^b^
nadirCD4+cell (range)	195.6 (1–577)	—	
currentCD4+cell (range)	445.0(22–1300)	—	
Log_10_ Viral load pre cART median (range)	4.79 (2.98–6.95)	—	
Duration of cART (months)	34 (12–218)	—	
Key drug (PI/NNRTI/INSTI)	207/36/82	—	

Data are presented as medians (ranges)

^a^ *t*-test,

^b^ Chi-squared test

Abbreviations: BMI, body mass index, cART, combination antiretroviral therapy, PI: protease inhibitor, INSTI, integrase inhibitor, NNRTI, non-nucleoside reverse-transcriptase inhibitor

### Measurement of TLs of peripheral blood leukocytes

DNA was extracted from peripheral blood leukocytes (PBLs) using a QIA amp Blood Mini kit (QIAGEN Inc., Tokyo, Japan). LTL was measured using real-time quantitative PCR as previously described [23] and expressed as a ratio to the copy number of the housekeeping gene, β2 microgloblin. A standard curve was prepared with genomic DNA of three HIV^-^ healthy volunteers by serial dilution. Genomic DNA extracted from a T-cell leukemia cell line, MOLT, was included as a reference in each PCR reaction to control for inter-assay variation. A negative control reaction without template was included in all PCR experiments. All samples were amplified in triplicate together with reference samples and negative controls. Data were expressed as a percentage of the telomere length of MOLT cells.

### MRI scans and WMH quantification

WMH was assessed in 184 HIV^+^ individuals by FRAIR imaging. All hyperintense subcortical lesions on FLAIR images (regardless of whether they occurred in white or subcortical grey matter) were labeled WMH. Segmentations were checked and reviewed by trained raters. At the time of reviewing MRI images, the raters were blinded to the patient’s identity and demographic factors and had not participated in the patients’ clinical care.

### Statistical analysis

We analyzed several covariates including markers of HIV infection, antiretroviral therapy, and social/environmental factors. In linear regression analyses to identify factors associated with LTL in HIV^+^ individuals, variables associated with TL length in univariate analyses (age, substance abuse, failure to achieve viral suppression after starting cART, cART regimen) were incorporated into multivariate models. Logistic regression analysis was used to identify variables associated with WMH. Variables associated with WMH by Mann-Whitney or Chi-squared tests were used in logistic regression models. First, each risk factor was modeled using univariate logistic regression. Subsequently, a multiple logistic regression model was constructed including all risk factors used in univariate logistic regression.

## Results

### Characteristics of study participants

Characteristics of the 325 HIV^+^ individuals and 147 healthy volunteers (frequency matched for age and sex) are shown in Table 1. All participants were Japanese. TL data were available for all participants. The majority (97%) of the HIV^+^ participants were male. The median age of HIV^+^ individuals was 39.0 years (range: 18–69 years) and was similar to that of HIV^-^ individuals (median: 39.5 years; range: 22–63 years) (p = 0.8281, *t*-test). No differences in age, BMI, or frequency of hypertension were observed between HIV^+^ and HIV^-^ participants. HIV^+^ participants were more likely to smoke, but this difference was not significant. All HIV^+^ participants were receiving cART and had median current CD4 T-cell counts of 445 cells/μL (range: 22–1300 cells/μL). HIV^+^ individuals had received cART for a median of 34 months (range: 12–218 months).

### Telomere lengths of PBLs

**Fig 1:**
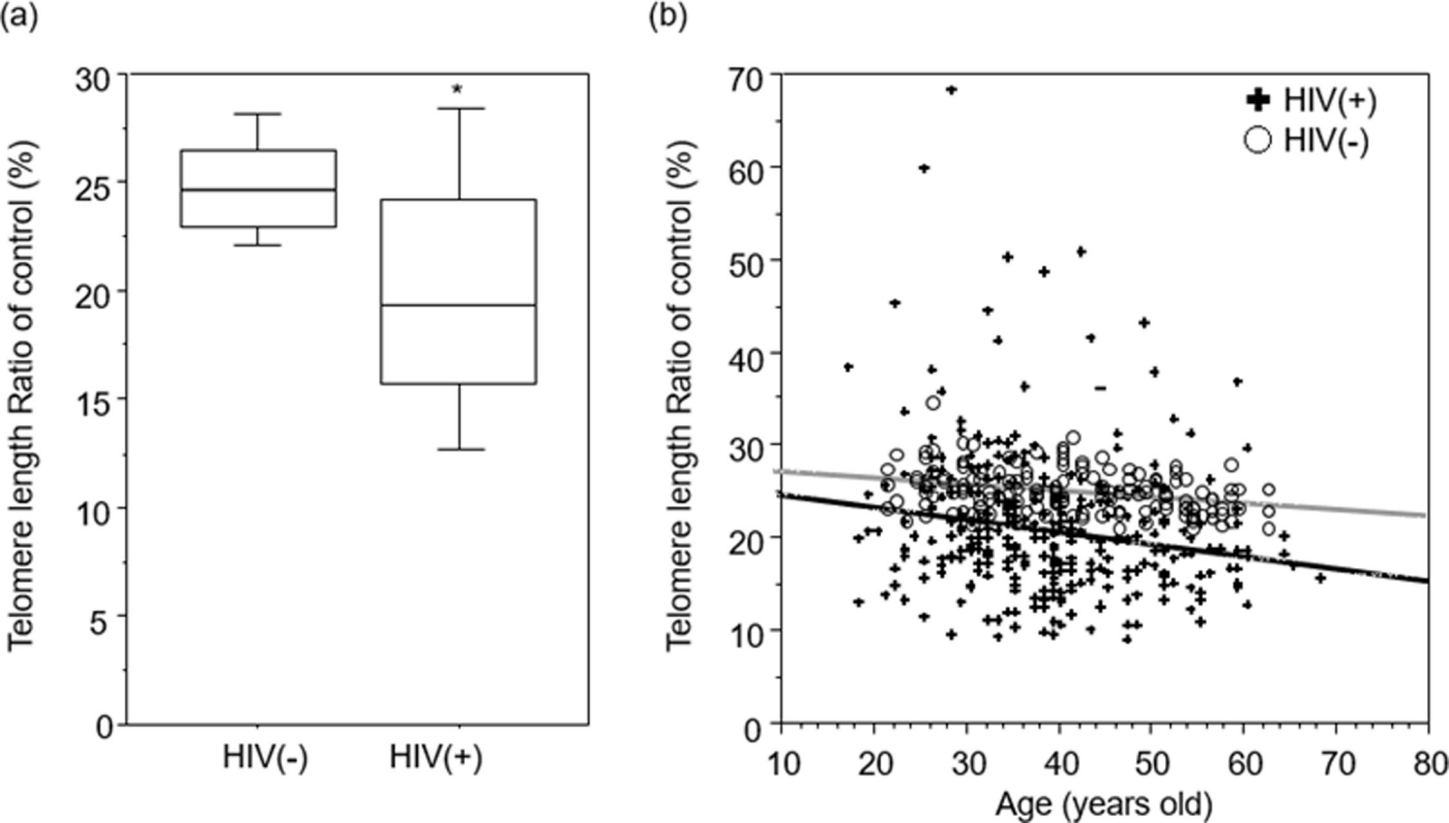
Comparison of TLs in HIV^+^ and HIV^-^ individuals. LTLs were measured using real-time quantitative PCR and expressed as a ratio to a housekeeping gene (T/S ratio). Data shown are normalized to an internal control. (a) Box plot showing differences in LTL between HIV^+^ and HIV^-^ individuals. * indicates p<0.0001 relative to HIV^-^ individuals by Mann-Whitney U-test. (b) Absolute LTL by age. Solid lines represent regression lines, black line represent HIV^+^ individuals (r = -0.134), and gray line represents HIV^-^ individuals (r = -0.069).

LTLs were significantly shorter in HIV^+^ individuals than in HIV^-^ individuals (Fig 1, P<0.0001, Mann-Whitney U-test). No associations were observed between TL length and age, sex, BMI, or smoking behavior in HIV^+^ and HIV^-^ individuals. Thus, multivariable analyses did not include these variables as confounders. There is a possibility that other confounding factors, such as the prevalence of substance abuse, could partially explain the difference in TL between HIV^+^ individuals and HIV^-^ controls.

There was an inverse association between advancing chronological age and LTL. In HIV^+^ individuals, the rate of LTL decrease with advancing chronological age was faster than in HIV^-^ individuals (Fig 1).

### Factors associated with LTL

**Fig 2:**
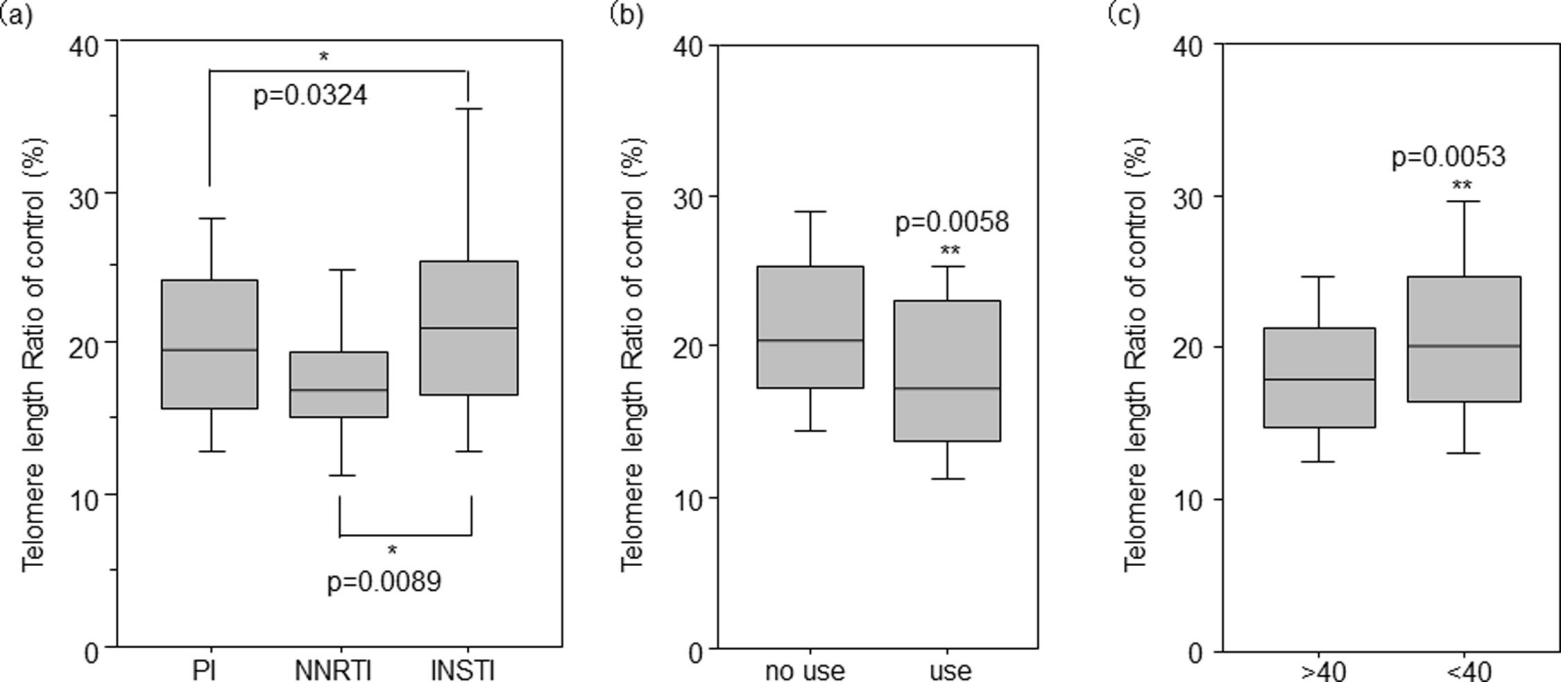
Factors associated with LTL in HIV^+^ individuals. LTL was measured using real-time quantitative PCR and expressed as a ratio to a housekeeping gene (T/S ratio). Data shown are normalized to an internal control. Differences in LTL are shown between (a) individuals treated with different cART regimens, (b) individuals with (use) or without (no use) a past history of substance use (cannabis, amphetamines, legal intoxicants, and 5-MeO-DIPT), and (c) individuals who achieved plasma HIV viral loads <40 RNA copies/mL (<40) or failed to achieve this level of control (>40) after 1 year of starting cART. * indicates statistical significance with P value obtained from the Bonferroni–Dunn test; ** indicates statistical significance with P value obtained from the Mann-Whitney U-test. Abbreviations: cART, combination antiretroviral therapy; NNRTI, nonnucleoside reverse transcriptase inhibitor; PI, protease inhibitor, INSTI, integrase strand transfer inhibitor.

To investigate the factors associated with LTL in HIV^+^ individuals, univariate regression analyses were performed for HIV^+^ individuals. Older age, past/present history of substance use, failure to achieve viral loads <40 RNA copies/mL within 1 year of initiating cART, and cART without INSTI were significantly associated with shorter TLs (Fig 2). These factors were all independently associated with shorter TLs in multivariate regression analyses adjusting for all other factors (Table 2). Neither plasma HIV viral load before cART, current CD4 count, CD4 nadir, nor duration of cART treatment showed any association with TL.

**Table 2:** Regression modeling of variables independently associated with telomere length in HIV+ individuals.

	Univariate linear regression analysis		Multivariate regression analysis	
	Regression coefficient	p value	Regression coefficient	p value
Age (years)	-0.184	0.0008*	-0.164	0.0379**
BMI	-0.022	0.6968		
Smoking (past)^a^	-0.079	0.251		
Smoking (Current)^a^	-0.029	0.678		
Hypertension^a^	-0.049	0.4706		
LDL-C (mg/dl)	-0.034	0.5829		
HDL-C (mg/dl)	0.038	0.539		
Substance abuse^a^	-0.26	0.0014*	-0.311	0.0001*
CD4 count (nadir) (/μl)	0.073	0.2007		
CD4 count(sampling) (/μl)	0.042	0.4502		
HIV-RNA (before cART)log	-0.075	0.236		
HIV-RNA>40^a^^,^^c^	-0.143	0.0122**	-0.17	0.0329**
cART^b^	-0.156	0.005*	-0.161	0.0425**
Duration of cART (months)	-0.001	0.9791		

Univariate and multivariate linear regression was used to identify factors associated with TL.

^a^ For categorical variables, “No” was scored 0 and “Yes” was scored 1.

^b^ cART, INSTI, NNRTI, and PI were scored 0, 1, and 2, respectively.

^c^ Failure to achieve HIV viral loads <40 RNA copies/mL within 1 year of initiating cART.

*indicates p values <0.01,

**indicates p values<0.05

Abbreviations: LDL-C, low density lipoprotein cholesterol, HDL-C, high density lipoprotein cholesterol, cART, combination antiretroviral therapy, NNRTI, non-nucleoside reverse transcriptase inhibitor, NRTI, nucleoside reverse transcriptase inhibitor, PI, protease inhibitor.

### Factors associated with WMH

Among the HIV^+^ participants, 184 individuals underwent brain MRIs. MRI findings are shown in Table 3. WMH was detected in 36 HIV^+^ individuals (18.9%). Older age (p <0.0001, Mann-Whitney U-test), shorter LTLs (p = 0.0006, Mann-Whitney U-test), hypertension (p = 0.0128, Chi-squared test), and carotid artery plaque (p = 0.0374, Chi-squared test) were significantly associated with WMH, while none of plasma viral load load before cART, current CD4 count, CD4 nadir, or length or type of cART exposure showed any association with WMH. In univariate logistic regression analysis, these four factors (older age, shorter LTLs, hypertension, and carotid artery plaques) were significantly associated with WMH. All four factors were included in multiple regression models, which showed that older age and shorter LTLs remained significant predictive factors associated with WMH (Table 4).

**Table 3:** Factors associated with leukoaraiosis in HIV^+^ individuals.

	Leukoaraiosis (-)	Leukoaraiosis (+)	P-value
Number (M/F)	146/3	33/3	0.1616
Age	40.7 ±0.77	52.4 ±1.72	<0.0001^a^
BMI	22.4 ±0.29	22.5 ±0.71	0.8808^a^
Telomere length	21.7 ±0.62	17.2 ±0.78	0.0006^a^
Smoking (past)	80(53.7%)	20(55.5%)	>0.9999^b^
(current)	54(36.2%)	13(36.1%)	0.7288^b^
Hypertension	18 (12.1%)	11(30.5%)	0.0091^b^
Carotid artery plaque (-/+)	21/27	1/12	0.0374^b^
LDL-C	101.3 ±2.7	108.0 ±5.3	0.2417^a^
HDL-C	47.9 ±1.67	43.1 ±1.84	0.4007^a^
Substance abuse	33(22.1%)	9(25%)	>0.9999^b^
Nadir CD4 number	155.9 ±11.0	129.5 ±24.7	0.2059^a^
HIV-RNA before cART(log10)	4.89 ±0.07	5.15 ±0.12	0.0982^a^
Duration of cART (month)	47.2 ±3.4	44.2 ±7.31	0.5070^a^
Key drug (PI/NNRTI/INSTI)	96/11/43	26/4/5	0.1664^b^

The data are presented as means ± standard errors for age, BMI, telomere length, LDL-C, HDL-C, nadir CD4 count, HIV RNA viral load, and duration of cART.

^a^ Mann-Whitney U test,

^b^ Chi-squared test

Abbreviations: BMI, body mass index, LDL-C, low density lipoprotein cholesterol, HDL-C, high density lipoprotein cholesterol, cART, combination antiretroviral therapy, INSTI, integrase inhibitor, PI, protease inhibitor, NNRTI,non-nucleoside reverse-transcriptase inhibitor

**Table 4:** Logistic regression modeling of factors independently associated with leukoaraiosis.

	Univariate logistic regression			Multivariate logistic regression		
	ORa	95%CI	P-value	ORa	95%CI	P-value
Age	1.124	1.075–1.175	<0.0001	1.167	1.040–1.309	0.0088*
Telomere length shortening	1.136	1.053–1.225	0.0009	1.23	1.03–1.45	0.023**
Hypertension	3.259	1.369–7.761	0.0076	1.285	0.189–8.719	0.7976
carotid artery plaquec	9.333	1.122–77.633	0.0388	9.175	0.63–133.5	0.1047

^a^ Odds ratios were calculated using univariate and multivariable logistic regression models.

^b^ older age, LTL shortening, hypertension, and carotid artery plaque were adjusted for in multiple logistic regression analysis.

^c^ Carotid artery plaque defined as carotid intima-media thickness (IMT) >1 mm, analyzed as a categorical variable.

*indicates p values <0.01,

**indicates p values<0.05

## Discussion

The precise mechanisms of shorter LTLs in HIV^+^ individuals remain unknown. Recently, shorter LTLs have been suggested to arise from changes in immune cell subsets in peripheral blood with respect to both frequency and differentiation state, rather than a global TL shortening among all leukocytes [24]. The proportion of more mature and differentiated innate and adaptive immune cells with shorter TLs increase among PBLs, which accounts for the reduced LTLs observed in HIV^+^ individuals. However, the biological significance of such changes still needs to be determined, and factors associated with shorter LTLs differed across studies, potentially due to the variety of backgrounds of study participants.

This study included 325 patients with HIV viral loads <40 RNA copies/mL who received cART for >12 months. We observed that shorter LTLs in HIV^+^ individuals were strongly associated with advancing age, cART without INSTI, failure to achieve viral control after initiation of cART, and substance use. In previous reports, cART and substance abuse have sometimes been associated with LTL shortening in HIV^+^ individuals [25, 26]. Antiretroviral drug-induced toxicity is an important complication of cART, particularly for regimens containing protease inhibitors (PIs). PIs increase the release of inflammatory cytokines, promote foam cell formation and induce macrophage apoptosis [27]. Zhang et al. demonstrated that raltegravir, an INSTI, was less toxic and avoided PI-induced upregulation of inflammatory cytokines [28]. Furthermore, poor viral control during cART induces low-level viremia, which is also associated with higher levels of inflammatory markers than sustained undetectable viremia. In substance users, drugs increase oxidative stress, which causes oxidative damage to telomeric DNA [29]; this damage may accelerate the rate of telomere shortening per cell division. Furthermore, a longitudinal study showed that individuals who received less toxic cART regimens (such as NRTI-sparing regimens) had the longest LTLs among individuals receiving cART who maintained an undetectable viral load for 2 years [30]. Our results are consistent with previous studies showing that oxidative stress and inflammation caused by antiretroviral drugs, low level viremia, or substance use may contribute to LTL shortening. However, we also obtained some contradictory results. We found no statistically significant difference in TL among HIV^+^ individuals with or without cART-induced mitochondrial toxicity. HIV patients with mitochondrial toxicity were found to have high expression of hTERT mRNA to maintain the length of telomeres [31]. However, this result was derived from a small study and did not consider the potential involvement of antiretroviral drugs, such as protease inhibitors, that inhibit telomerase activity. Thus, these seemingly discordant results may in fact be reconcilable.

However, we found no association between shorter LTLs and active smoking or multiple characteristics of the metabolic syndrome (high body mass index (BMI), high LDL-cholesterol, and low HDL cholesterol level), all of which contribute to LTL shortening in HIV^-^ individuals. Other cross-sectional or longitudinal studies also showed that smoking or other metabolic factors had no significant effect on LTL shortening in HIV^+^ individuals [30, 32, 33]. Our results and these reports suggest that these factors may be less important in HIV^+^ individuals and are masked by other factors associated with HIV infection. The regression coefficients in univariate and multivariate analysis in this study was relatively small, so it is possible that other pivotal factors that remain unknown are associated with shorter LTL.

Our results showed that leukoaraiosis was associated with age in HIV^+^ individuals. Older HIV^+^ individuals had higher WMH than younger ones. Several studies have investigated the association between WMH and HIV infection. Seider et al. showed that a relationship between age and greater WMH was present only in HIV^+^ individuals [34]. In general, leukoaraiosis occurs as a result of disruption of small penetrating arteries in the brain; the condition is common past age 80, but occurs minimally before age 60 [35]. During HIV infection, vascular damage may be caused by HIV itself, by some antiretroviral drugs, by metabolic complications such as dyslipidemia, insulin resistance, and diabetes, by low-grade chronic systemic or local inflammation, or by co-infections such as hepatitis C or cytomegalovirus. Microbial translocation, including fungal translocation from the gut, also contributes to chronic inflammation and immune activation during HIV infection, and a potential link between fungal translocation and neurocognitive impairment has been demonstrated [36]. Thus, vascular damage continues to occur in HIV^+^ individuals in spite of cART, and leukoaraiosis is observed at younger ages and progresses at a higher rate than in uninfected individuals. In our study, shorter LTL was independently associated with leukoaraiosis. Inflammation and oxidative stress might mediate the association between LTL and leukoaraiosis, because they are involved in both vascular damage and LTL shortening. Therefore, LTL shortening could predict changes in brain structure and function. One might use LTL shortening as an indicator to manage modifiable risk factors for white matter damage, such as metabolic complications, before the development of significant functional impairments. As HIV^+^ individuals age, it will become more important to investigate the influence of HIV infection, comorbid conditions, and age on brain structure and function.

This study had several limitations. As our study had a cross-sectional design, we were able to assess associations between WMH and LTL, but could not describe the predictive value of LTL as a biomarker of the aging brain. Longitudinal studies would allow for more direct observation of the combined effects of age, HIV, and other risk factors on WMH.

Second, the sample size was small, limiting statistical power especially in some of the smaller subgroup analyses. Third, we did not perform MRI scans on HIV^-^ controls for comparison, and we used only a visual rating of WMHs. Thus, we could not understand the etiology of leukoaraiosis, nor determine how HIV and age-related factors influenced white matter damage. Further larger and preferably longitudinal studies are needed to confirm our preliminary findings and to determine whether LTL can be used as a predictive biomarker of HIV-associated, age-related brain disease.

To our knowledge, this is the first report to demonstrate an association between leukoaraiosis and short LTLs in HIV^+^ individuals. Identifying early signs of TL shortening HIV^+^ individuals and stopping this process is important to avoid structural and functional damage to the brain.

## Conclusions

LTL shortening was independently associated with leukoaraiosis in HIV^+^ individuals, and was also associated with age, viral control during cART, use of PIs, and substance use. These factors may mask the influence of other HIV disease or environmental parameters. Effective viral control and less toxic cART can help improve outcomes among HIV^+^ individuals.

## References

[1] Lagathu C, Cossarizza A, Béréziat V, Nasi M, Capeau J, Pinti M. Basic science and pathogenesis of ageing with HIV: potential mechanisms and biomarkers. AIDS. 2017; 31: S105–S119. 10.1097/QAD.0000000000001441 28471941

[2] Hornsby PJ. Telomerase and the aging process. Exp Gerontol. 2007; 42: 575–581. 10.1016/j.exger.2007.03.007 17482404 PMC1933587

[3] Barnes RP, Fouquerel E, Opresko PL. The impact of oxidative DNA damage and stress on telomere homeostasis. Mech Ageing Dev. 2018 3 28 pii: S0047-6374(18)30052-6. 10.1016/j.mad.2018.03.013 29604323 PMC6162185

[4] Kimura M, Gazitt Y, Cao X, Zhao X, Lansdorp PM, Aviv A. Synchrony of telomere length among hematopoietic cells. Exp Hematol 2010; 38: 854–859. 10.1016/j.exphem.2010.06.010 20600576 PMC3142672

[5] Gardner JP, Kimura M, Chai W, Durrani JF, Tchakmakjian L, Cao X, et al Telomere dynamics in macaques and humans. J Gerontol A Biol Sci Med Sci 2007; 62: 367–374. 10.1093/gerona/62.4.367 17452729

[6] von Zglinicki T, Martin-Ruiz CM. Telomeres as biomarkers for ageing and age-related diseases. Curr Mol Med. 2005; 5(2):197–203. 15974873 10.2174/1566524053586545

[7] D'Mello MJ, Ross SA, Briel M, Anand SS, Gerstein H, Paré G. The Association Between Shortened Leukocyte Telomere Length and Cardio-Metabolic Outcomes: A Systematic Review and Meta-Analysis. Circ Cardiovasc Genet. 2014 11 18 pii: CIRCGENETICS.113.000485. 10.1161/CIRCGENETICS.113.000485 25406241

[8] Eguchi K, Honig LS, Lee JH, Hoshide S, Kario K. Short telomere length is associated with renal impairment in Japanese subjects with cardiovascular risk. PLoS One. 2017; 12(4):e0176138 10.1371/journal.pone.0176138 28441430 PMC5404870

[9] Nielsen BR, Linneberg A, Bendix L, Harboe M, Christensen K, Schwarz P. Association between leukocyte telomere length and bone mineral density in women 25–93 years of age. Exp Gerontol. 2015; 66: 25–31. 10.1016/j.exger.2015.04.004 25868397

[10] Torres RA, Lewis W. Aging and HIV/AIDS: pathogenetic role of therapeutic side effects. Lab Invest. 2014;94(2):120–8. 10.1038/labinvest.2013.142 24336070 PMC4144856

[11] von Zglinicki T, Serra V, Lorenz M, Saretzki G, Lenzen-Grossimlighaus R, Gessner R, Risch A, Steinhagen-Thiessen E. Short telomeres in patients with vascular dementia: an indicator of low antioxidative capacity and a possible risk factor? Lab Invest. 2000; 80(11):1739–47. 11092534 10.1038/labinvest.3780184

[12] Herrmann M, Pusceddu I, März W, Herrmann W. Telomere biology and age-related diseases. Clin Chem Lab Med. 2018;56(8):1210–1222. 10.1515/cclm-2017-0870 29494336

[13] Beirne C, Delahay R, Hares M, Young A. Age-related declines and disease-associated variation in immune cell telomere length in a wild mammal. PLoS One. 2014 9 30;9(9):e108964 10.1371/journal.pone.0108964 25268841 PMC4182606

[14] Comandini A, Naro C, Adamo R, Akbar AN, Lanna A, Bonmassar E, Franzese O. Molecular mechanisms involved in HIV-1-Tat mediated inhibition of telomerase activity in human CD4(+) T lymphocytes. Mol Immunol. 2013;54(2):181–92. 10.1016/j.molimm.2012.12.003 23287597

[15] Zanet DL, Thorne A, Singer J, Maan EJ, Sattha B, Le Campion A, Soudeyns H, Pick N, Murray M, Money DM, Côté HC; CIHR Emerging Team Grant on HIV Therapy and Aging: CARMA. Association between short leukocyte telomere length and HIV infection in a cohort study: No evidence of a relationship with antiretroviral therapy. Clin Infect Dis. 2014;58(9):1322–32. 10.1093/cid/ciu051 24457340

[16] Haddow LJ, Dudau C, Chandrashekar H, Cartledge JD, Hyare H, Miller RF, Jäger HR. Cross-sectional study of unexplained white matter lesions in HIV positive individuals undergoing brain magnetic resonance imaging. AIDS Patient Care STDS. 2014;28(7):341–9. 10.1089/apc.2013.0230 24785779 PMC4074759

[17] Pantoni L. Cerebral small vessel disease: from pathogenesis and clinical characteristics to therapeutic challenges. Lancet Neurol. 2010;9:689–701. 10.1016/S1474-4422(10)70104-6 20610345

[18] Fazekas F., Schmidt R. and Scheltens P. Pathophysiologic mechanisms in the development of age-related white matter changes of the brain. Dement Geriatr Cogn Disord. 1998;9 Suppl 1:2–5. 10.1159/000051182 9716237

[19] Lin J, Wang D, Lan L, Fan Y. Multiple Factors Involved in the Pathogenesis of White Matter Lesions. Biomed Res Int. 2017;2017:9372050 10.1155/2017/9372050 28316994 PMC5339523

[20] Debette S, Markus HS. The clinical importance of white matter hyperintensities on brain magnetic resonance imaging: systematic review and meta-analysis. BMJ. 2010 7 26;341:c3666 10.1136/bmj.c3666 20660506 PMC2910261

[21] Raina A, Zhao X, Grove ML, Bressler J, Gottesman RF, Guan W, Pankow JS, Boerwinkle E, Mosley TH, Fornage M. Cerebral white matter hyperintensities on MRI and acceleration of epigenetic aging: the atherosclerosis risk in communities study.Clin Epigenetics. 2017 2 14;9:21 10.1186/s13148-016-0302-6 eCollection 2017 28289478 PMC5310061

[22] Yuen T, Brouillette MJ, Fellows LK, Ellis RJ, Letendre S, Heaton R, et al Personalized Risk Index for Neurocognitive Decline Among People With Well-Controlled HIV Infection. J Acquir Immune Defic Syndr. 2017;76(1):48–54. 10.1097/QAI.0000000000001466 28797021 PMC12077807

[23] Cawthon RM. Telomere measurement by quantitative PCR. Nucleic Acids Res. 2002 5 15;30(10):e47 10.1093/nar/30.10.e47 12000852 PMC115301

[24] Kaushal S, Landay AL, Lederman MM, Connick E, Spritzler J, Kuritzkes DR, Kessler H, Levine BL, St Louis DC, June CH. Increases in T cell telomere length in HIV infection after antiretroviral combination therapy for HIV-1 infection implicate distinct population dynamics in CD4+ and CD8+ T cells. Clin Immunol. 1999 7;92(1):14–24. 10.1006/clim.1999.4726 10413649

[25] Saberi S, Kalloger SE, Zhu MMT, Sattha B, Maan EJ, van Schalkwyk J, et al Dynamics of leukocyte telomere length in pregnant women living with HIV, and HIV-negative pregnant women: A longitudinal observational study. PLoS One. 2019;14(3):e0212273 10.1371/journal.pone.0212273 30840638 PMC6402636

[26] Lai S, Heaphy CM, Rizzo AJ, Celentano DD, Gerstenblith G, Li J, et al Cocaine use may induce telomere shortening in individuals with HIV infection. Prog Neuropsychopharmacol Biol Psychiatry. 2018;84(Pt A):11–17. 10.1016/j.pnpbp.2018.01.015 29410247 PMC5880737

[27] Wang X, Liao D, Lin PH, Yao Q, Chen C. Highly active antiretroviral therapy drugs inhibit in vitro cholesterol efflux from human macrophage-derived foam cells. Lab Invest. 2009;89(12):1355–63. 10.1038/labinvest.2009.85 19770838 PMC2787635

[28] Zhang X, Cao R, Liu R, Zhao R, Huang Y, Gurley EC, Hylemon PB, Pandak WM, Wang G, Zhang L, Li X, Zhou H. PLoS One. 2014 3 13;9(3):e90856 10.1371/journal.pone.0090856 24625618 PMC3953206

[29] Oikawa S, Kawanishi S. Site-specific DNA damage at GGG sequence by oxidative stress may accelerate telomere shortening. FEBS Lett. 1999;453(3):365–8. 10405177 10.1016/s0014-5793(99)00748-6

[30] Montejano R, Stella-Ascariz N, Monge S, Bernardino JI, Pérez-Valero I, Montes ML, et al Impact of Nucleos(t)ide Reverse Transcriptase Inhibitors on Blood Telomere Length Changes in a Prospective Cohort of Aviremic HIV-Infected Adults. J Infect Dis. 2018;218(10):1531–1540. 10.1093/infdis/jiy364 29912427

[31] Li M, Foli Y, Amakye NY, Klein T, Selvaraj S, Lu L, et al Antiretroviral therapy-induced toxicity is associated with increased mRNA expression of telomerase. J Clin Pharmacol. 2015;55(10):1119–24. 10.1002/jcph.520 25903721 PMC4558235

[32] Araújo ML, Duarte W, Oliveira ACP, Gascón MRP, Fonseca LAM, Paiva RMA, et al Is the telomere length associated with neurocognitive disabilities in HIV-1-infected subjects? Rev Inst Med Trop Sao Paulo. 2018;60:e16 10.1590/s1678-9946201860016 29694602 PMC5956918

[33] Stella-Ascariz N, Montejano R, Rodriguez-Centeno J, Alejos B, Schwimmer C, Bernardino JI, et al Blood Telomere Length Changes After Ritonavir-Boosted Darunavir Combined With Raltegravir or Tenofovir-Emtricitabine in Antiretroviral-Naive Adults Infected With HIV-1. J Infect Dis. 2018;218(10):1523–1530. 10.1093/infdis/jiy399 29982509

[34] Seider TR, Gongvatana A, Woods AJ, Chen H, Porges EC, Cummings T, Correia S, Tashima K, Cohen RA. Age exacerbates HIV-associated white matter abnormalities. J Neurovirol. 2016 4;22(2):201–12. 10.1007/s13365-015-0386-3 26446690 PMC4783252

[35] Hopkins RO, Beck CJ, Burnett DL, Weaver LK, Victoroff J, Bigler ED. Prevalence of white matter hyperintensities in a young healthy population. J Neuroimaging. 2006;16(3):243–51. 10.1111/j.1552-6569.2006.00047.x 16808826

[36] Hoenigl M, de Oliveira MF, Pérez-Santiago J, Zhang Y, Morris S, McCutchan AJ, et al (1→3)-β-D-Glucan Levels Correlate With Neurocognitive Functioning in HIV-Infected Persons on Suppressive Antiretroviral Therapy: A Cohort Study. Medicine (Baltimore). 2016 3;95(11):e3162 10.1097/MD.0000000000003162 26986173 PMC4839954

